# Flare: An FPGA-Based Full Precision Low Power CNN Accelerator with Reconfigurable Structure

**DOI:** 10.3390/s24072239

**Published:** 2024-03-31

**Authors:** Yuhua Xu, Jie Luo, Wei Sun

**Affiliations:** School of Electronics and Information Technology (School of Microelectronics), Sun Yat-sen University, Guangzhou 510275, China; xuyh55@mail2.sysu.edu.cn (Y.X.); luoj269@mail2.sysu.edu.cn (J.L.)

**Keywords:** FPGA accelerator, convolutional neural networks, full precision, design space exploration, dynamic partial reconfiguration

## Abstract

Convolutional neural networks (CNNs) have significantly advanced various fields; however, their computational demands and power consumption have escalated, posing challenges for deployment in low-power scenarios. To address this issue and facilitate the application of CNNs in power constrained environments, the development of dedicated CNN accelerators is crucial. Prior research has predominantly concentrated on developing low precision CNN accelerators using code generated from high-level synthesis (HLS) tools. Unfortunately, these approaches often fail to efficiently utilize the computational resources of field-programmable gate arrays (FPGAs) and do not extend well to full precision scenarios. To overcome these limitations, we integrate vector dot products to unify the convolution and fully connected layers. By treating the row vector of input feature maps as the fundamental processing unit, we balance processing latency and resource consumption while eliminating data rearrangement time. Furthermore, an accurate design space exploration (DSE) model is established to identify the optimal design points for each CNN layer, and dynamic partial reconfiguration is employed to maximize each layer’s access to computational resources. Our approach is validated through the implementation of AlexNet and VGG16 on 7A100T and ZU15EG platforms, respectively. We achieve an average convolutional layer throughput of 28.985 GOP/s and 246.711 GOP/s for full precision. Notably, the proposed accelerator demonstrates remarkable power efficiency, with a maximum improvement of 23.989 and 15.376 times compared to current state-of-the-art FPGA implementations.

## 1. Introduction

CNNs [1] have demonstrated remarkable prowess in feature abstraction, a pivotal phase within the realm of image processing. They find wide application in various domains including medical image recognition, autonomous driving, and beyond [2,3,4]. However, the intensive multiply-accumulate (MAC) operations required by CNNs pose a challenge for general CPUs to achieve acceptable processing latency, emphasizing the urgency for dedicated accelerators.

GPUs currently dominate the accelerator design landscape due to their ease of use and high performance capabilities. However, their substantial power requirements render them unsuitable for deployment in low-power scenarios. Application-specific integrated circuits (ASICs) offer high throughput with minimal power consumption, but their significant development costs limit their viability for widespread consumer use. Additionally, the fixed circuit structure of ASICs makes them susceptible to obsolescence as neural network architectures evolve. In contrast, FPGAs offer a compelling solution by striking a balance between power consumption, computational throughput, and flexible programming [5]. This versatility makes FPGAs an ideal platform for deploying neural networks, as they can adapt to evolving network architectures while meeting the power constraints of various applications.

Despite the inherent advantages of FPGAs, deploying CNNs on FPGA platforms can be a time-consuming endeavor, even for seasoned experts. To expedite this process, many researchers turn to HLS tools to automatically generate Verilog hardware description language (HDL) code from C/C++ code [6]. However, while HLS tools offer a quicker path to implementation, they often fall short of achieving optimal resource consumption and operation scheduling compared to manually crafted designs by experienced FPGA designers [7]. Consequently, a significant gap exists between the actual performance achieved and the theoretical limits imposed by the external memory bandwidth and computational capacity of FPGAs, particularly when dealing with full-precision networks, as depicted in the roofline model [8].

In this paper, we introduce Flare, an FPGA-based full precision low power CNNs accelerator with reconfigurable structure, designed to address existing barriers. Our work makes the following key contributions:

- We introduce a design space exploration (DSE) model aimed at evaluating various accelerator configuration parameters accurately, including resource consumption and processing latency. This model enables us to significantly reduce the development period by providing precise performance estimations across multiple criteria.
- We propose a vector dot product with variable length to unify the computation pattern of convolutional and fully connected layers in CNNs. The fine-grained length, in conjunction with DSE, facilitates a balanced allocation of computational resources and off-chip memory bandwidth.
- We adopt a run-time reconfiguration method to maximize each layer’s access to computational resources. The extra time overhead introduced by reconfiguration flows can be offset by faster processing time, thereby reducing overall inference latency.

The remainder of this paper is organized as follows: Section 2 introduces the related works on FPGA-based CNN accelerators, Section 3 presents the details of our proposed design space exploration algorithm, which is used to guide the structure design process, and then the resulting accelerator framework is given in Section 4. Section 5 presents a comprehensive evaluation and discussion on experiment results. Finally, Section 6 concludes.

## 2. Related Works

As shown in Algorithm 1, the inherent implementation of convolutional layers (CLs) often relies on nested loops, resulting in severe data dependence, non-parallel execution, and limited data-reuse. Consequently, the primary objectives in designing a CNN accelerator are twofold: to efficiently acquire a larger volume of data within the constraints of the off-chip memory bandwidth and to promptly process the acquired data.

To simultaneously reduce memory-access time and computation latency, various network-lightening methods have been proposed. Network pruning aims to set less important weights to zero, thereby reducing the computational overhead associated with multiplication operations [9]. However, the control and transformation required for sparse computation may introduce additional processing latency and yield limited gains, potentially increasing error rates. Alternatively, network quantization [10,11,12,13] aims to represent original 32-bit float weights with fewer bits, allowing for more efficient data transfer and reduced resource utilization. Despite the consensus that full precision weights are unnecessary for certain applications, the gradient of the quantization function required for backward propagation cannot be computed [14], limiting its application in high-accuracy scenarios.
**Algorithm 1:** Nested Loop Implementation for Convolutional Layer
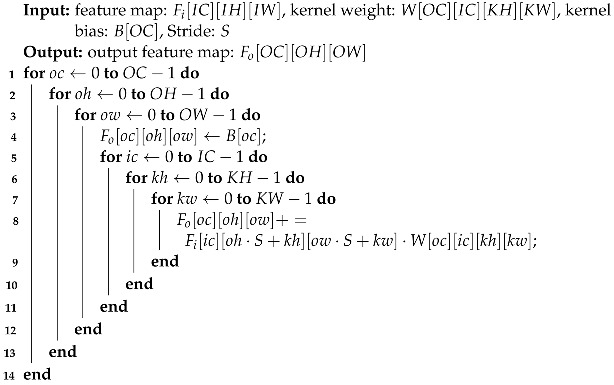


Instead of directly executing MAC operations as depicted in Algorithm 1, alternative accelerator designs based on transformations have been investigated. Modern processors accommodate general matrix multiplication (GEMM) libraries to accelerate basic linear algebra calculations. Zhang et al. first introduced this idea to FPGA-based CNN design by using the “im2col” method to transform the calculation of CLs into matrix multiplication through reshaping and packing [15]. However, the “im2col” + GEMM approach requires significant on-chip memory to store the transformed image matrix, making it impractical for mid-low end FPGA platforms. The Winograd algorithm [16] aims to reduce the number of multipliers by employing elaborate computation transformations, which can be beneficial for small convolutional kernels. However, the overhead associated with complex transformation functions and additional adders may render it unsuitable for CLs with larger kernel sizes. Fast Fourier transform (FFT) is a classical algorithm used to accelerate convolution calculations and has been adopted in CNN accelerators [17]. However, the FFT length needs to be adjusted for each layer, and additional computations for complex numbers, as well as time for (inverse) transformation, must also be taken into account.

The low computational efficiency of Algorithm 1 primarily arises from the nested loops’ data dependence. This challenge can be partially mitigated by employing sophisticated optimization techniques like polyhedral models and loop tiling methods, which are well-established in compiler technology. Zhang et al. applied these methodologies to direct the Verilog HDL code generation process using HLS tools, supplemented by a DSE model to pinpoint the optimal optimization parameters required to attain their performance objectives [6]. However, a significant limitation of their work is that when the overall structure of the CNN is divided into multiple layers, the optimization parameters for each layer may not lead to overall optimality due to incompatibility between sub-solutions, a problem that was recently pointed out by researchers [18]. Additionally, their paper does not report whether the predicted DSE data matches experimental results, making it difficult to assess the effectiveness of their scheme. Moreover, many papers struggle to implement all layers on FPGA simultaneously to achieve a pipeline network structure. However, this approach necessitates meticulous resource allocation to each layer to prevent pipeline idling due to workload imbalances, and few of these papers [19] consider the structure of each layer from the perspective of coordinating all layers.

It’s evident that burst length significantly impacts bandwidth utilization during data transfer, and the non-sequential memory access of Algorithm 1 results in inefficient data movement. To address this, data rearrangement [20] is proposed to reorder data based on the order of access. Some works [6,21,22,23] focus solely on accelerating CLs, which account for the vast majority of computation, however, the processing latency for Fully connected layer (FCLs) is never negligible due to the limited bandwidth.

## 3. Design Space Exploration

Mapping strategies for deploying networks onto FPGA platforms can significantly impact system performance. Therefore, having a guiding model for structure optimization is crucial for efficiently exploring this design space. In this section, we begin by characterizing the utilization and processing latency of dot product modules on given vector length and parallelism levels. Subsequently, we establish a parallelism allocation method that considers the bandwidth of off-chip memory to search for feasible input and output parallelism configurations with the minimize need for data caching. Finally, we evaluate all potential configurations and select the optimal scheme that exhibits the lowest processing latency, thus ensuring optimal performance on the FPGA platform.

### 3.1. Dot Product with Variable Length

From a functional perspective, CLs are utilized for extracting image features, whereas FCLs serve classification purposes. However, when delving into the specific calculations involved, both types of layers can be conceptually equated to the dot product of two vectors, as exemplified in Equation (1), and the symbol “⊗” denotes the convolution operation.

For vectors with vecLen elements, it is straightforward to observe that the processing latency can be expressed by Equation (2). Here, the term 1 represents the latency associated with multipliers, while $\left\lceil \log_{2}vecLen\right\rceil$ denotes the latency of the adder tree, and the final boolean expression ensures the utilization of at least one adder. Notably, the iteration interval of this sub-module is 1, enabling the circuit to receive and process new data every clock cycle in the pipeline.

Leveraging this characteristic, we can partition a larger dot product with a size of LEN into smaller segments of size vecLen. An additional adder is incorporated to accumulate the outputs of the “dotProduct” sub-module. The overall latency is derived in Equation (3), where the first term represents the initial iteration latency, the second term accounts for the additional iterations required, and the final term determines the necessity of an extra adder.

(1) $$\left[\begin{array}{ccc} f_{00} & f_{01} & f_{02}\\ f_{10} & f_{11} & f_{12}\\ f_{20} & f_{21} & f_{22} \end{array}\right]⊗\left[\begin{array}{ccc} w_{00} & w_{01} & w_{02}\\ w_{10} & w_{11} & w_{12}\\ w_{20} & w_{21} & w_{22} \end{array}\right]=\left[\begin{array}{ccccccccc} f_{00} & f_{01} & f_{02} & f_{10} & f_{11} & f_{12} & f_{20} & f_{21} & f_{22} \end{array}\right]\cdot \left[\begin{array}{c} w_{00}\\ w_{01}\\ w_{02}\\ w_{10}\\ w_{11}\\ w_{12}\\ w_{20}\\ w_{21}\\ w_{22} \end{array}\right]$$

(2) $$Ltc1=1+\left\lceil \log_{2}vecLen\right\rceil +boolean\left(vecLen==1\right)$$

(3) $$Ltc2=Ltc1+\left(\left\lceil \frac{LEN}{vecLen}\right\rceil -1\right)+boolean\left(\left\lceil \frac{LEN}{vecLen}\right\rceil >1\right)$$

In addition to processing latency, the number of “dotProduct” instances supported by the FPGA platform, which is determined by resource consumption, is also an essential consideration during the accelerator design phase. Table 1 presents the resource utilization of basic operators “adder” and “mul”, and Equation (4) can then be utilized to compute the resource consumption of a dot product with vecLen elements. Suppose the system is concurrently running *N* “dotProduct” instances with size vecLen, then the resource utilization can be expressed as shown in Equation (5), where the second term accounts for the additional *N* adders required to accumulate the outputs of each “dotProduct” instances.

(4) $$\begin{array}{c} \left[\begin{array}{c} vec\_LUT,vec\_FF,vec\_DSP \end{array}\right]=vecLen\cdot \left[\begin{array}{c} mul\_LUT,mul\_FF,mul\_DSP \end{array}\right]+\\ \left(vecLen-1\right)\cdot \left[\begin{array}{c} adder\_LUT,adder\_FF,adder\_DSP \end{array}\right] \end{array}$$

(5) $$\begin{array}{c} \left[\begin{array}{c} N\_LUT,N\_FF,N\_DSP \end{array}\right]=N\cdot \left[\begin{array}{c} vec\_LUT,vec\_FF,vec\_DSP \end{array}\right]+\\ N\cdot \left[\begin{array}{c} adder\_LUT,adder\_FF,adder\_DSP \end{array}\right] \end{array}$$

### 3.2. Bandwidth-Aware Parallelism Allocation Scheme

**Figure 1:**
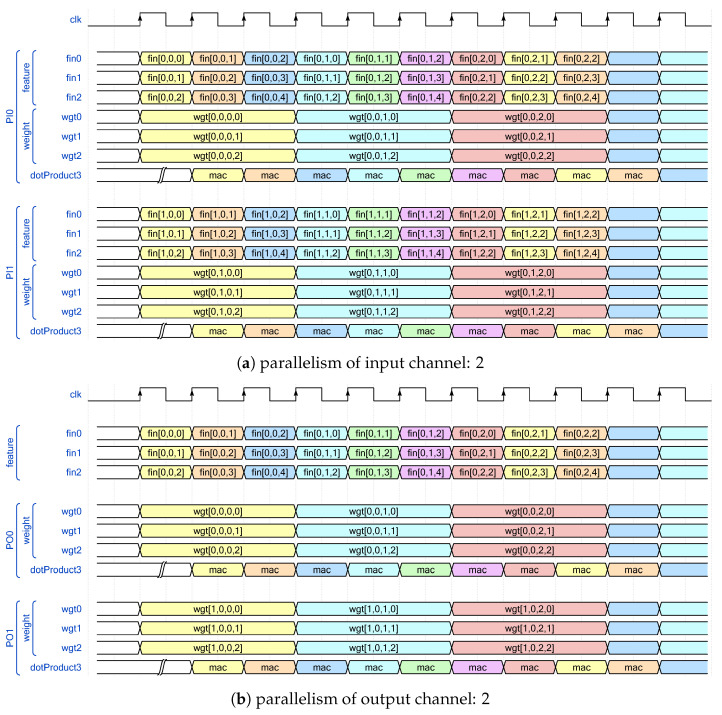
Timing diagram of two possible parallelism allocation scheme with IW = 5, OW = 3, KH = KW = 3, S = 1, vecLen = 3 and N = 2. (**a**) Assign all parallelism to input channel. (**b**) Assign all parallelism to output channel.

In theory, once the length of the dot product vecLen and the total degree of parallelism *N* have been specified, the approximate processing latency is determined accordingly. For instance, the used clocks of Algorithm 1 can be characterized by Equation (6), where the numerator represents the total MAC operations of a given CL, and the denominator denotes the number of MACs handled per clock cycle. However, this assumes a system with sufficient off-chip memory bandwidth. Two straightforward examples with vecLen = 3 and N = 2 in Figure 1 can be referenced to better illustrate how bandwidth impacts system performance.

(6) $$Ltc=\frac{OC\cdot OH\cdot OW\cdot IC\cdot KH\cdot KW}{vecLen\cdot N}$$

Figure 1a depicts a scenario where all the parallelism N = 2 are assigned to input channel. In this configuration, two sets of independent input feature map data Fin and corresponding weight data Wgt are required. Specifically, for every three clock cycles, IW · N = 10 Fin data and KW · N = 6 Wgt data are necessary. Conversely, Figure 1b assigns all the parallelism to the output channel, necessitating only IW= 5 Fin data and KW · N = 6 Wgt data. This approach effectively leverages data reuse of Fin across the output channel to mitigate the significant bandwidth demands, making it a more favorable solution for FPGA platforms with limited off-chip bandwidth.

**Figure 2:**
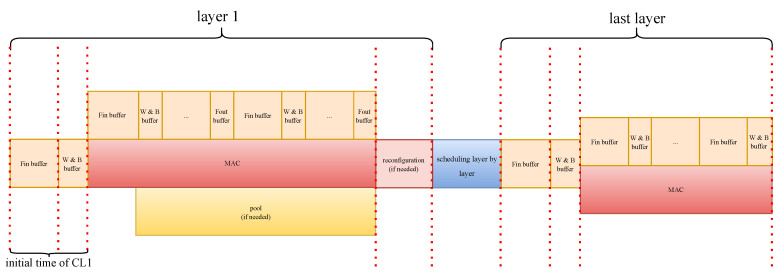
Ideal timing of the overall structure.

While allocating all parallelism *N* to the output channel may initially appear as a rational decision, the revised latency estimator defined in Equation (7) highlights that improper selection of parallelism parameters for the input and output channels, denotes as PI and PO (while N = PI · PO), respectively, can lead to wasted clock cycles due to ceiling operations. We defer a discussion of this aspect to the concluding part of this section and instead concentrate on exploring the existence of a viable parallelism allocation scheme given the available data per clock (DPC). Concurrently, we must contemplate the worst-case scenario where no viable allocation scheme may exist due to the constraints imposed by limited off-chip memory bandwidth. To address this challenge, we employ a caching strategy where Fin and Wgt data are buffered prior to MAC operations. This approach allows MAC operations to seamlessly read data from the buffer while simultaneously caching new data. The ideal timing sequence of our architecture is depicted in Figure 2. Consequently, our focus now shifts to identifying the parallelism allocation scheme with the minimum initial time, as outlined in Algorithm 2.

(7) $$Ltc=\left\lceil \frac{KW}{vecLen}\right\rceil \cdot \left\lceil \frac{IC}{PI}\right\rceil \cdot \left\lceil \frac{OC}{PO}\right\rceil \cdot OH\cdot OW\cdot KH$$

(8) $$DPC=\frac{1024\cdot BW(Gbps)}{Freq(MHz)\cdot DW(bits)}$$

The term “row_clocks” mentioned in the first line of Algorithm 2 represents the number of clocks required to process a row of data from Fin. The first component, $\left\lceil \frac{KW}{vecLen}\right\rceil$, specifies the minimum number of clocks needed to process every segment of KW data within Fin. The second component, OW, indicates that Fin can be conceptually divided into OW set of KW data each. The timing diagram depicted in Figure 1 provides a a clearer understanding of this concept. Lines 2 to 6 of the algorithm are used to traverse and search for all potential parallelism allocation schemes.

To narrow the search space of the caching scheme while introducing minimal buffering time, we cache at least KH rows of data for each parallel channel of Fin, along with 1 row of Wgt for PI and PO channels in advance, and then assess the need for caching additional fin_rows and wgt_rows of Fin and Wgt, respectively, as indicated in the 7th and 14th lines of Algorithm 2. The “if” statement from line 9 to 13 calculates the number of clocks required to process the currently cached Fin data.
**Algorithm 2:** Parallelism Allocation Algorithm
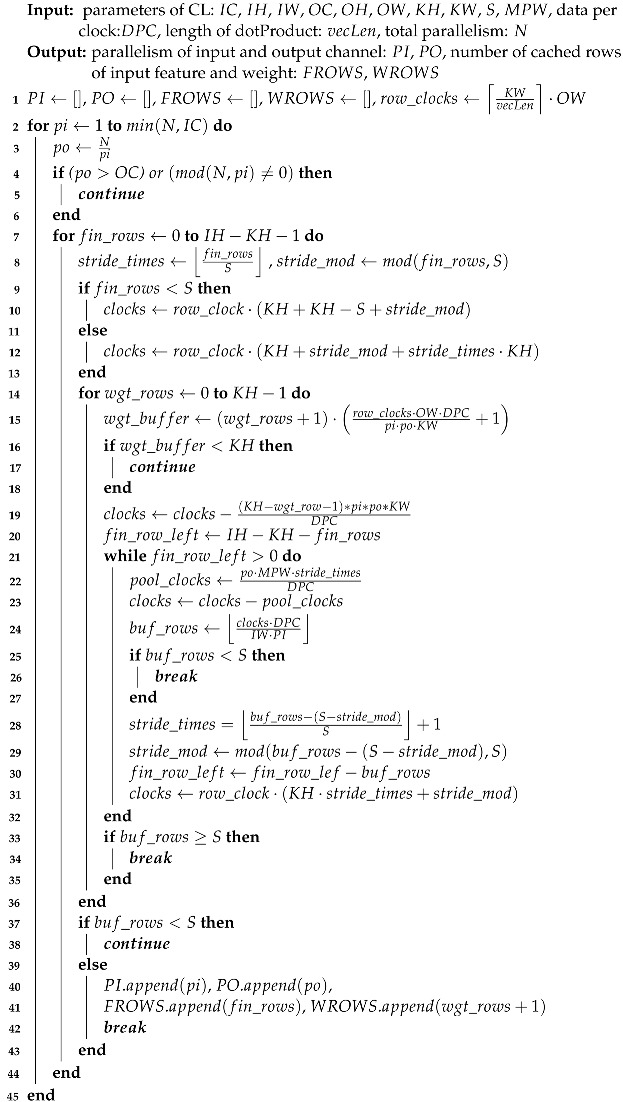


The statement from line 15 to 18 calculates the number of extra rows of Wgt needed to be cached to ensure that the time for caching the remaining Wgt data can be covered by the processing of already cached data. Given that at least KH rows of Fin for PI channels and all Wgt data for PI and PO channels are cached, the only consideration is whether the next stride S rows of Fin for PI channels can be cached or not. This judgment is executed as part of the statement from line 19 to 32. It’s important to note that lines 22 and 23 assume a scenario where PO · MPW pool layer data is generated for every *S* rows of Fin, and all this data is written directly to off-chip memory without on-chip buffering, while this represents a worst-case scenario unlikely to occur in a real structure

In essence, Algorithm 2 strives to minimize the initial time of the CL and ensures that subsequent MAC operations within the pipeline do not stall due to data unavailability. To accomplish this objective, we opt to process the row vector of Fin for two primary reasons: firstly, a large burst length can be used to cache the consecutive data of Fin to increase the utilization of bandwidth; secondly, processing Fin at the row level is more suitable when the length of dotProduct vecLen does not exceed the width of the convolution kernel KW, this enables more granular parallelism allocation to the input and output channels.

### 3.3. Decision of the Best Design Point

Based on our preceding analysis, the overall flowchart of our proposed DSE algorithm comprises three pivotal components. Initially, we establish the values of vecLen and *N* by taking into account resource consumption factors, as expounded in lines 1 to 6 of Algorithm 3. Subsequently, we identify all viable parallelism allocation strategies that offer minimal initial time, with consideration given to off-chip bandwidth limitations, as delineated in line 7. Lastly, we estimate the processing latency associated with each current design point to ascertain the one that exhibits the lowest latency, as outlined in lines 8 to 14. This comprehensive approach ensures the optimal design of FPGA-based CNN accelerators, tailored to meet specific resource and performance requirements.

Two additional factors warrant attention. While high resource utilization can indeed contribute to reduced processing latency, it can also give rise to wire congestion, potentially leading to timing violations. In our work, we propose an empirical guideline that restricts feasible design points to those with LUT utilization below 0.7. Furthermore, the extra reconfiguration time is also considered. Specifically, if the design point for the preceding layer has a processing latency lower than the result of DSE plus the reconfiguration time, the former design point should be remained.
**Algorithm 3:** Overall flow of the proposed DSE algorithm
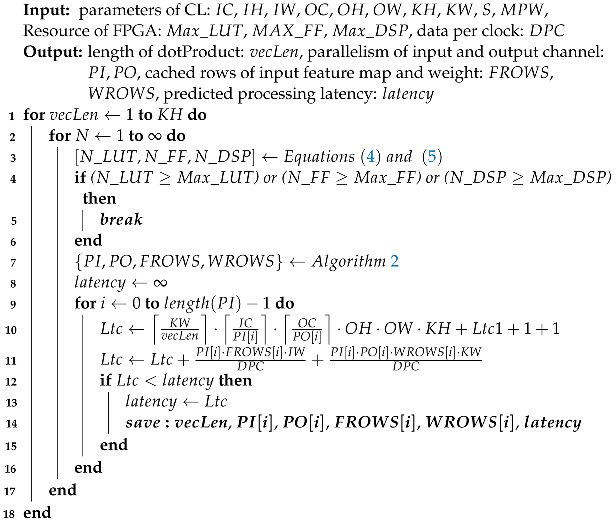


## 4. Architecture of Proposed CNN Accelerator

**Figure 3:**
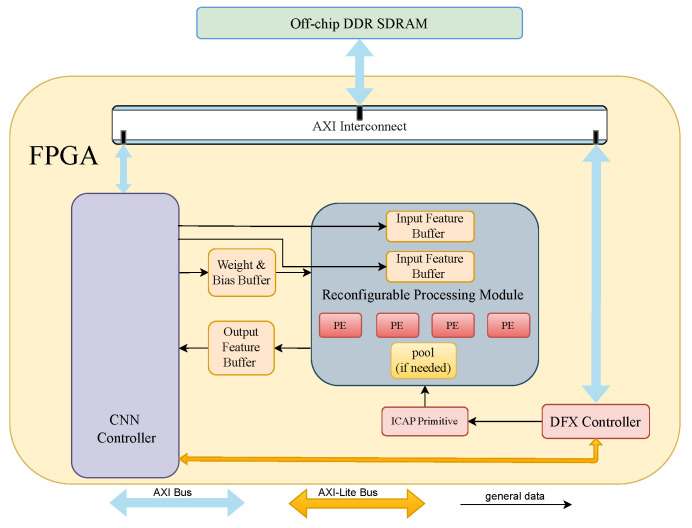
The overall architecture of our proposed CNN accelerator.

Our proposed accelerator structure, as depicted in Figure 3, leverages on-chip memory caching with data reuse to mitigate the demand for bandwidth, and additionally, it dynamically reconfigures the processing element layer by layer to ensure each layer can access the maximum resources of the FPGA platform. To automatically update the circuit required by the next layer after completing the computation of a CNN layer, we employ the Dynamic Function Exchange (DFX) Controller IP and the Internal Configuration Access Port (ICAP) primitive to manage the bitstream files for each layer. This streamlines the process of loading the necessary bitstream files onto the FPGA. The subsequent sub-sections provide detailed insights into additional circuit structures integral to this design.

### 4.1. Integration with Design Space Exploration

In the preceding section, our aim was to determine the optimal configurations for each layer. However, transitioning from abstract parameters to specific circuit structures remains challenging. To provide a clearer understanding of integrating the output of the DSE model—comprising parameters such as the length of the dot product (vecLen), input channel parallelism (PI), output channel parallelism (PO), and the required cached rows of Fin and Wgt data (FROWS and WROWS)—within a specific FPGA design, we approach this section from a high-level perspective.

**Figure 4:**
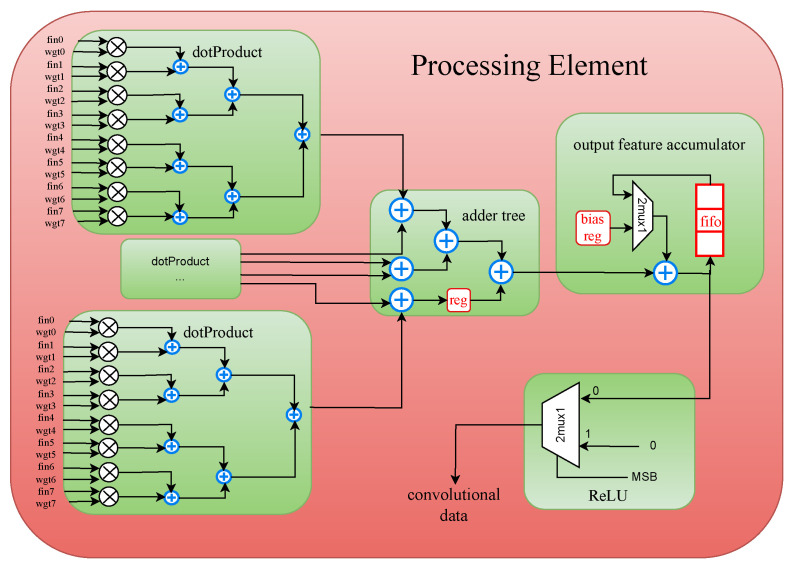
Structure of processing element.

The parameter PO signifies the quantity of processing element (PE) modules in Figure 3, each responsible for generating an output feature map channel. Within each PE module, illustrated in Figure 4, the count of “dotProduct” sub-modules should correspond to the value of PI, signifying that PI channels of input feature map data are processed simultaneously. Similarly, the vector length handled by each “dotProduct” module should align with vecLen. Additionally, the number of input feature buffer modules in Figure 4 matches PI, ensuring dedicated buffers for each input channel, thereby enhancing on-chip memory bandwidth.

In scenarios where a CL is followed by a pooling layer, there will be PO sub-modules of “Comparer” or “average” within the corresponding max pool or average pool module. These modules rely on values derived from DSE and are thus incorporated into the “Reconfigurable Processing Module” to ensure alignment of each layer’s structure with the expected values.

The parameters FROWS and WROWS do not manifest in specific circuit structures; instead, they serve as logical conditions indicating whether the initial required Fin and Wgt data have been cached in the CNN controller module.

### 4.2. Processing Element

The processing element (PE), as illustrated in Figure 4, serves as the fundamental building block of a CNN accelerator, tasked with efficiently executing a significant number of MAC operations.

**Figure 5:**
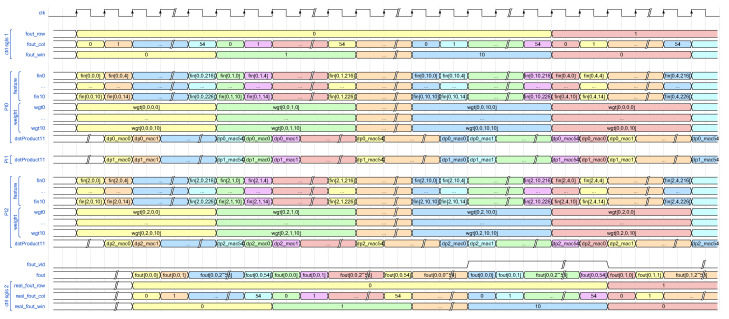
An example timing diagram of processing element.

Consider the first CL of AlexNet with PI = 3, PO = 1, KH = 11, and vecLen = 11 as an example, and refer to the corresponding timing diagram illustrated in Figure 5. Initially, 3 “dotProduct” modules are implemented to perform MAC operations for three input feature map channels, denoted as groups “PI0”, “PI1” (for brevity, sub-groups of feature and weight are omitted), and “PI2”.

Additional signals, including “fout_row”, “fout_col”, and “fout_win” within group “ctrl sig1”, are introduced to denote the coordinates of the expected output data, facilitating control and debugging processes. Specifically, “fout_win” indicates the row of the convolutional kernel presently engaged, reflecting our treatment of the row vector of Fin as the primary processing unit. The assertion of the “fout_vld” signal, indicating the validity of output data, occurs only when “fout_win” reaches KH - 1. Conversely, when “fout_win” equals 0, it signifies that the PE is currently processing the first row of the convolutional kernel. In this scenario, the equation simplifies to fout = dp0_mac + dp1_mac + dp2_mac + bias. Otherwise, the equation is adjusted to fout = dp0_mac + dp1_mac + dp2_mac + fout. The preceding three fixed terms, “dp0_mac”, “dp1_mac”, and “dp2_mac”, represent the MAC operation results of the 3 “dotProduct” modules within the PE, subsequently summed in the subsequent adder tree submodule. The final term embodies either the bias value necessary for the ongoing computation or the result of the previous row’s computation.

Considering processing latency, the “fout_vld” signal and the “ctrl sig2” group delineate the validity of the output feature map data and its precise position. The implementation of the “ReLU” module involves straightforward examination of the most significant bit of “fout” when the data is valid.

### 4.3. Pooling Layer

**Figure 6:**
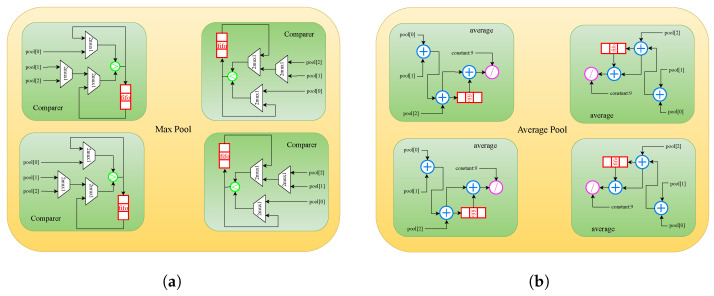
Structure of pool layer. (**a**) max pool with PO = 4; (**b**) average pool with PO = 4.

Pooling layers serve a crucial role in reducing the size of the output feature map and enhancing the generalization capabilities of CNNs through down-sampling operations. Two widely employed pooling operations are max pooling and average pooling, with their respective structures depicted in Figure 6a,b. As implied by their names, the output of a pooling layer represents the maximum or average value of the data within each pooling window.

**Figure 7:**
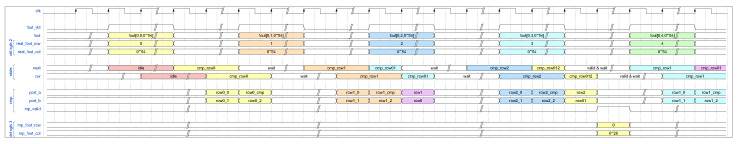
An example timing diagram of max pooling layer.

The structure for a pooling layer is relatively straightforward to implement. As such, we provide an example timing diagram of a max pooling layer with 3×3 pooling windows and a stride S of 2 as a case study, illustrated in Figure 7. The state machine transitions from the “idle” state to the “cmp_row0” state upon receiving the first valid row data from “fout”, subsequently, two comparison stages are employed to process the row data. The first stage involves comparing every two adjacent data points, followed by comparing the output of the comparator with the remaining data in a row pooling window in the next stage. A FIFO is employed to buffer the output of the comparator in the second stage. Upon processing the second valid row data of “fout”, an additional comparison operation is executed in the “cmp_row01” state, and the result is cached using the same FIFO. Similarly, after transitioning through the “cmp_row2” and “cmp_row012” states, the first row data of the max pooling layer is obtained. It is noteworthy that the data labeled “row2” is treated as “row0” due to the stride.

Due to the adequate timing gap between the arrival of valid row data in “fout”, a notable advantage of our proposed pooling structure is that the processing latency can be compensated for by the PE module, as depicted in Figure 2.

### 4.4. Data Buffer

**Figure 8:**
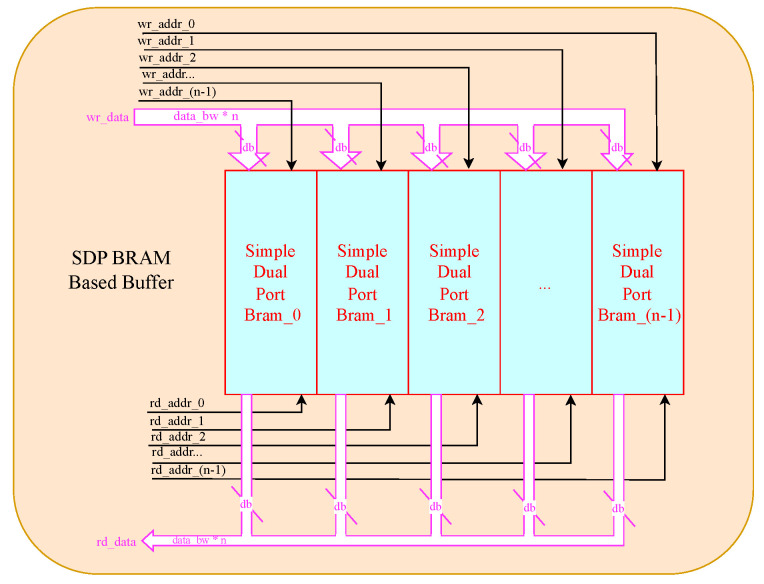
Structure of proposed data buffer.

Data reuse significantly mitigates the heavy demand for off-chip DDR bandwidth, while a longer burst length enhances bandwidth utilization. Therefore, the design of the data buffer structure needs to consider both these factors carefully. In our work, the input feature map, weights, and bias data buffer primarily facilitate data reuse, whereas the output feature map data buffer primarily supports data rearrangement. To accomplish this design, we employ the simple dual-port block RAM (SDP Bram) with a size of 32 bits × 512 as the fundamental unit for constructing the data buffer, as depicted in Figure 8, with each SDP BRAM having an individual address to ensure fine-grained data access.

In our system design, each CL is furnished with PI data buffers tailored for caching Fin data. Each buffer consists of 16 SDP Bram units, enabling access to KW (not exceeding 11) data within a single clock cycle. The quantity of Fin data buffers varies based on the variable PI, thus necessitating their integration into the reconfigurable processing module, as depicted in Figure 3. Conversely, the weight and bias buffer remain static, comprising 32 SDP Brams. This decision stems from the observation for output of DSE model that $\frac{32\cdot OW}{KW}$ consistently exceeds PI · PO, ensuring adequate time for weight data refreshing. Concerning the output feature map buffer, we employ 32 and 64 SDP Bram units for the XC7A100T and ZU15EG platforms, respectively. This allocation is guided by two primary considerations. Firstly, the DSE model affirms sufficient time for transmitting “fout” data to on-chip DDR memory, even in the absence of a data buffer. Secondly, the allocated storage capacity adequately accommodates the initial Fin data required for subsequent CL or FCL layers. Consequently, this allocation significantly reduces the initialization time for subsequent layers, leveraging the relatively high bandwidth of on-chip memory.

**Figure 9:**
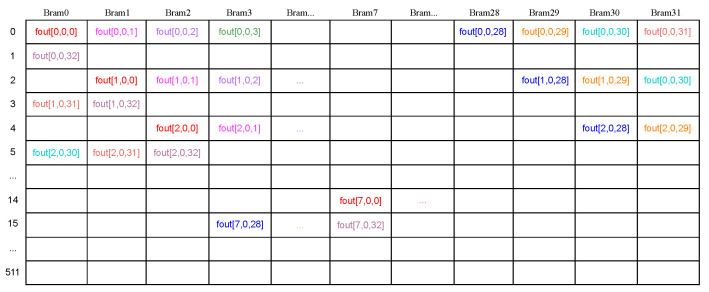
Data arrangement of output feature map.

Data rearrangement serves as a key technique for enhancing burst length during data transfer, a topic extensively explored in numerous research papers. However, it’s essential to consider the additional latency introduced by online data rearrangement, a challenge we address from three key perspectives. Firstly, the row vectors of Fin naturally exhibit continuity, alleviating the need for extensive rearrangement efforts. Secondly, we perform offline data rearrangement before storing weight data into DDR, leveraging the predictability of access patterns by DSE in advance. Finally, despite the PE modules generating PO data of “fout” for every valid clock, our proposed buffer structure seamlessly transforms the column vector of parallel output channels into row vectors for each output channel, as illustrated in Figure 9. Notably, the data sharing the same color signifies that they were generated during the same clock cycle. Given that the number of SDP Brams in the “fout” buffer exceeds all feasible PO values for every layer, all PO data can be cached into the buffer within a single clock cycle by writing to the corresponding SDP Bram. For instance, the data “fout[po,0,col]” will be written to SDP Bram mod(po + col, 32), with the address being po$\cdot \left\lceil \frac{OW}{32}\right\rceil$ for data from the PE modules or po$\cdot \left\lceil \frac{MPW}{32}\right\rceil$ for data from the pooling layers. With careful address generation, “fout” data can be efficiently transferred to DDR in row vector mode.Succinctly put, data rearrangement involves discrete writing and continuous reading operations.

The buffer’s storage capacity is insufficient to accommodate the entirety of the feature map or weight data involved. Consequently, we employ control logic resembling that of a FIFO. Essentially, new data can overwrite old data only when the buffer is not at full capacity.

## 5. Experimental Results

In the preceding two sections, we delved into the intricacies of identifying the optimal design parameters and structure for our proposed framework. This section presents experimental results to offer a comprehensive assessment of our work. The experimental FPGA platforms utilized include the Xilinx XC7A100T-2FGG484I, equipped with 1 GB DDR3 SDRAM, and the Xilinx XCZU15EG-2FFVB1156I, featuring 4 GB DDR4 SDRAM. These platforms boast maximum off-chip bandwidths of 3.125 GB/s and 18.75 GB/s, respectively. We utilized Vivado 2023.1 for synthesizing the accelerator design.

### 5.1. Results of Design Space Exploration

The rarely reported metrics on resource consumption and processing latency, as predicted by the DSE model, are provided to assess the alignment between actual performance and predicted values. Table 2 and Table 3 represent the results of DSE for AlexNet [24] and VGG16 [25], respectively.

The “Original” section provides the structural parameters of each layer. For VGG16, the specifications such as the size of the convolution kernel, stride, and padding remain constant at 3×3, 1, and 1, respectively. We have omitted these specific parameters to save space. The column “[L, PI, PO]” represents the key design points provided by DSE, indicating the length of the “dotProduct” vecLen and the parallelism for input and output channels. In the case of FCLs, “L” is fixed to the value of DPC, as expressed in Equation (8). This suggests that the performance of FCLs is constrained by the bandwidth of off-chip memory rather than the resources of the FPGA platform. The data in the column “[fr, wr]” denotes the minimum rows of input feature map, weight, and bias data that should be cached in advance, corresponding to the FROWS and WROWS output of Algorithm 2. We omit these two parameters for VGG16 implemented on the XC7A100T platform for the same purpose, which is consistently fixed at [3, 2] for CLs and [-, 1] for FCLs.

It can be observed that the length of the “dotProduct”, denoted as L, equals the width of the convolution kernel for both networks on both platforms. This alignment helps minimize the processing latency for a row vector of Fin, as indicated by row_clocks in the first line of Algorithm 2. From the results of PI and PO, it is apparent that parallelism is more preferentially assigned to the output channel for platforms with low off-chip bandwidth, aligning with our previous analysis that a larger PI will lead to a higher demand for bandwidth. The vast majority of “fr” and “wr” are close to the height of the convolution kernel and 2, respectively. This implies that the initial time of the layer can be achieved at a low level and imposes a small requirement on the on-chip memory resource for data caching.

Based on the processing latency item “Ltc”, it is clear that the total time needed for FCLs in AlexNet surpasses that of CLs, despite most MAC operations occurring in CLs. This trend persists with VGG16, where the processing time proportion of FCLs remains notable in the overall processing duration. Consequently, strategies exclusively targeting convolutional layer acceleration, as observed in certain studies, are deemed inadequate.

### 5.2. Resource Consumption and Latency in FPGA Implementations

After synthesizing and implementing the system circuit guided by DSE, we obtained the actual hardware resource utilization and latency, which are presented in Table 4.

For layers sharing identical values for L · PI · PO among different design points, it is foreseeable that processing latency will scale with the total number of MAC operations across nested loops. Consequently, certain design points with optimal configuration parameters may not yield latency improvements due to additional latency introduced by reconfiguration. In such instances, maintaining the original structure is advisable to avoid unnecessary complexity. Considering this, the most notable distinction between Table 4 and the preceding tables lies in the more uniform structures among CLs. Essentially, given the relatively minor latency increases in some layers, maintaining a consistent structure across similar layers proves beneficial in minimizing the overall impact on processing time. Additionally, the reconfiguration time “Rcfg” required for VGG16 is only 2.122 ms and 13.099 ms on two platforms, respectively, compared to the reconfiguration time of 5.769 ms and 20.158 ms for AlexNet. This suggests that a uniform structure for the convolutional kernel may be more suitable for implementation on the FPGA platform.

The column “Ltc” in Table 2 and Table 3 indicates that the expected processing latency for FCLs closely matches the time required to transfer weight data from DDR to FPGA, a correspondence validated by real experiments. This alignment is understandable because FCLs typically involve abundant weight data but relatively fewer MAC operations, rendering them more dependent on off-chip memory bandwidth rather than FPGA computational resources. Consequently, the error rate associated with processing latency, denoted as “Ltc_e” in Table 4, is approximately zero. For CLs, the “Ltc_e” does not exceed 0.146% and 0.172% for the two platforms, respectively. Upon analysis of debugging waveforms, we discovered that a significant portion of this error stems from non-immediate handshaking and non-valid data during burst transfers of the AXI bus. These issues introduce additional latency and can adversely affect overall performance. In the case of non-first layers, the Fin data serves as the output of a preceding layer and is partially cached in on-chip memory. The uncertainty associated with AXI bus transfers can be mitigated by retrieving data from the cache, resulting in a substantial reduction in “Ltc_e”. This observation underscores the critical importance of efficient data management and caching strategies in minimizing latency errors in CNN acceleration.

The term “lut” specifically denotes the LUT utilization of the PE within the discussed context, while the LUT utilization of additional modules, such as the AXI interconnect module and CNN controller module, is not addressed in this analysis. This focus aims to evaluate the accuracy of the DSE model. Upon conducting a simple calculation, it was determined that the maximum error rate for “lut” is 8.169% and 4.354% for two FPGA platforms, respectively. This variation can be attributed to the fact that, apart from the circuits responsible for MAC operations, such as the “dotProduct” and adder tree, additional controlling logic is required to manage data flow, thereby increasing the LUT utilization.

### 5.3. Simulation as Case Study

**Figure 10:**
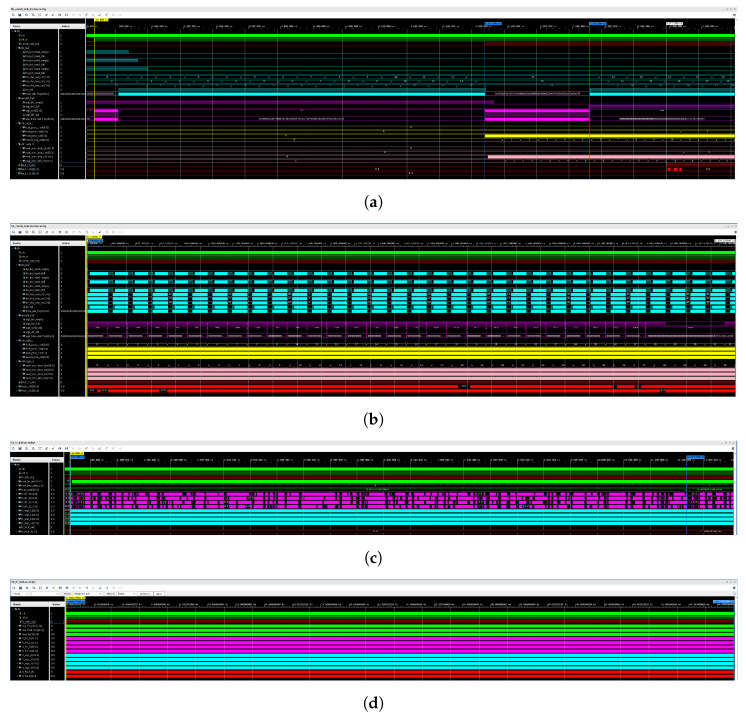
Simulation waveforms for the first CL and FCL of AlexNet on the 7A100T platform. (**a**) the beginning part of simulation waveforms for the first CL of AlexNet on the 7A100T platform. (**b**) the complete simulation waveforms for the first CL of AlexNet on the 7A100T platform. (**c**) the beginning part of simulation waveforms for the first FCL of AlexNet on the 7A100T platform. (**d**) the complete of simulation waveforms for the first FCL of AlexNet on the 7A100T platform.

To validate the accuracy of our DSE model, we provide simulation waveforms of the first CL and the first FCL of AlexNet on the XC7A100T platform, as illustrated in the following figures. Figure 10a,c depicts the initial segments of the waveforms for the CL and FCL processing stages, respectively, while Figure 10b,d shows the complete simulation waveforms for these two layers.

In Figure 10a, following the de-assertion of the reset signal, the weight buffer module, denoted as “weight_buf”, initiates caching of 72 clocks of weight data, calculated as $\frac{PI\cdot PO\cdot wr\cdot 12}{8}$. Here, we choose 12 instead of KW because every 4 floating-point numbers, or 128 bits, can form the required 256-bit width of the weight buffer module. This design decision is informed by the access pattern of weight data obtained from the DSE results. Additionally, before storing weight data into DDR, we append an extra 0 to each row of the convolutional kernel to ensure a width of 12, as discussed in the data rearrangement part of Section 4. Because the rearrangement of weight data can be accomplished offline, it does not introduce extra processing latency. Subsequently, the input feature map buffer module “fin_buf” caches 13 rows for each of the three input channels, corresponding to PI values, through polling. Upon caching the specified initial weight and input feature map data, “conv0_calc_ing” is asserted to indicate the initiation of data calculation by the PE module. During PE module computation, the weight buffer module first reads the remaining weight data from DDR, filling the direct buffer module. This strategy optimizes caching by prioritizing weight data, which exhibits significant data sharing characteristics, allowing for efficient on-chip caching of required weight data in a shorter time. The remaining processing time is then allocated for input feature map data caching. The control signals “ctrl_sgls_1” and “ctrl_sgls_2” serve the same purpose as the corresponding signals in the PE module processing timing diagram depicted in Figure 5, indicating the coordinate of the current processing and obtained data. It is important to note that, to prioritize the display of more essential signals, the weight and feature map data involved in computation are not shown in the simulation graph.

Figure 10b presents an overview of the CL computation process. The data buffers efficiently utilize DDR bandwidth by continuously fetching data for processing as long as storage are not “full”, thereby minimizing the waste of DDR bandwidth. Moreover, the persistent assertion of the “fout_ch_vld” signal signifies our circuit’s ability to maintain an efficient pipelined operation with an iteration interval of II = 1. Additionally, the processing latency of the first CL in AlexNet is approximately 5.330 ms, closely matching the DSE model’s estimate of 5.334 ms, demonstrating the successful application of the DSE model to the CLs.

Combining the simulation waveforms of the FCL in Figure 10c,d, we observe that, due to limitations in off-chip memory bandwidth, each clock cycle can only support single parallelism computation with a vector length of 4. It takes approximately 11.54 ms to complete the computation of the vector dot product with a length of 9216. The computation of the first fully connected layer is finalized at around 47.1746 ms, aligning closely with the DSE model’s estimated processing latency of 47.174 ms. This reaffirms the capability of the DSE model developed in this study to provide relatively accurate predictions of the calculation latency for each CNN layer, thus offering valuable guidance for accelerator system design.

### 5.4. Compare with Previous Works

A comparison of our accelerator with previous works is presented in Table 5, focusing on key metrics. The “Device” item specifies the experimental platform, which the maximum attainable computation resource is then determined. Because all the FPGA platform used in this comparison are from AMD Xilinx, we have excluded the “XC” prefix, denoting Xilinx Commercial, to conserve space. “Precision” indicates the quantization scheme, with examples such as our use of 32-bit full precision floating point and Huang’s adoption of 8-bit and 16-bit fixed point for weight and internal temporary data, respectively [19]. “Bandwidth” denotes the maximum off-chip memory bandwidth. These three factors crucially impact accelerator performance. “Latency” measures processing time for CLs and/or overall layers, and we have used “(C)” and “(O)” to distinguish between the data respectively. “Performance” is the primary evaluation criterion, typically in GFLOP/s for floating-point accelerators and GOP/S for fixed-point ones. Additionally, “Power” reflects power consumption. Efficiency relative to performance ensures a fair comparison across power, logic cells, and DSPs.

When compared with the Nvidia GTX 1080Ti, all FPGA-based accelerators demonstrate lower power consumption, though they fail to achieve higher throughput performance, with the exception of Qu’s work [26]. Particularly noteworthy is our achievement of the lowest power consumption among existing works. Despite doubling the system processing frequency on the XC7A100T platform compared to the initial attempt of deploying a full precision network [6], we only achieve approximately half the performance. This performance gap can be attributed to the fact that CLs are more sensitive to the computational resources of the FPGA platform. However, this gap disappears when the network is deployed on the ZU15EG, which offers significantly more computation resources.

While accelerators employing quantified weights generally promise improved performance over their full precision counterparts, it is noteworthy that [27] demonstrated the poorest performance among similar quantified works, even falling short of our VGG16 implementation deployed on the ZU15EG platform. This under performance may be attributed to their reliance on CPU assistance for Softmax function computation and intricate data rearrangement, resulting in notable latency. Additionally, [20] exhibits a substantial performance gap compared to other quantified works and only slightly outperforms our works on ZU15EG. This disparity primarily arises from their utilization of HLS for system design. However, current automatic code generation tools struggle to efficiently utilize off-chip memory bandwidth, leading to higher resource consumption compared to manually optimized code.

The studies conducted by [19,23,26] demonstrate commendable performance. However, when assessing power efficiency, our accelerators deployed on the 7A100T platform do not surpass the performance of these three works. Additionally, there exists a twofold power efficiency gap at worst case for the ZU15EG platform. In terms of logic cell and DSP efficiency, targeting full precision networks inherently results in lower efficiency compared to fixed-point implementations. Nevertheless, considering that our deployed network operates at full precision, this twofold gap is deemed acceptable, as there are numerous optimizations available for quantified fixed-point numbers as opposed to floating-point numbers. For example, with 16-bit quantified weights, the processing time for fully connected layers is at least halved, and multiplication or addition can be achieved using just a single DSP. With meticulous system design, DSPs can achieve faster data computation at twice the system frequency [28]. Hence, considering these aspects, our work can be regarded as achieving performance comparable to floating-point processing to some extent.

## 6. Conclusions

In this paper, we present a novel low-power CNN accelerator characterized by a reconfigurable architecture operating at full precision. Our approach leverages vector dot product to streamline the computational framework across convolutional layers and fully connected layers. Furthermore, we incorporate row-level pipeline streaming and employ a data buffer strategy to minimize the complexities associated with data rearrangement. We develop a design space exploration model to efficiently identify optimal design points for each layer. Remarkably, our model achieves high accuracy, with predicted latency error rates of within 0.146% and 0.172% for the XC7A100T and ZU15EG platforms, respectively. Moreover, our proposed framework outperforms previous works in terms of power consumption, demonstrating a maximum improvement of 23.989 and 15.376 times compared to current state-of-the-art FPGA implementations. This positions our work as a potential candidate for deployment in power-constrained applications. Looking ahead, the Verilog HDL code of our method demonstrates high regularity, paving the way for future automation in code generation through template mapping.

## Figures and Tables

**Table 1:** Resource Utilization of Adder and Mul.

Operator	XC7A100T				ZU15EG			
	LUT	FF	DSP	Latency	LUT	FF	DSP	Latency
adder	322	135	1	1	279	135	2	1
mul	277	135	1	1	277	135	1	1
total	63,400	126,800	135	-	341,280	682,560	3528	-

**Table 2:** DSE results for Alexnet.

Layer	Original			XC7A100T				ZU15EG			
	Fin	Wgt	[S,P]	[L,PI,PO]	[fr,wr]	lut	Ltc (ms)	[L,PI,PO]	[fr,wr]	lut	Ltc (ms)
CL0	[3,227,227]	[64,3,11,11]	[4,0]	[11,3,2]	[13,8]	0.623	5.334	[11,3,13]	[12,8]	0.699	0.557
CL1	[64,27,27]	[192,64,5,5]	[1,2]	[5,1,14]	[5,2]	0.661	16.330	[5,5,17]	[5,2]	0.692	1.896
CL2	[192,13,13]	[384,192,3,3]	[1,1]	[3,1,24]	[3,2]	0.680	7.788	[3,13,11]	[3,2]	0.699	0.888
CL3	[384,13,13]	[256,384,3,3]	[1,1]	[3,3,8]	[3,2]	0.680	10.383	[3,11,13]	[3,2]	0.699	1.183
CL4	[256,13,13]	[256,256,3,3]	[1,1]	[3,3,8]	[3,2]	0.680	6.977	[3,11,13]	[3,2]	0.699	0.812
FCL0	9216	[4096,9216]	-	[4,-,-]	[-,1]	0.038	47.174	[16,-,-]	[-,1]	0.039	7.864
FCL1	4096	[4096,4096]	-	[4,-,-]	[-,1]	0.038	20.966	[16,-,-]	[-,1]	0.039	3.494
FCL2	4096	[1000,4096]	-	[4,-,-]	[-,1]	0.038	5.119	[16,-,-]	[-,1]	0.039	0.853

**Table 3:** DSE results for VGG16.

Layer	Original		XC7A100T			ZU15EG			
	Fin	Wgt	[L,PI,PO]	lut	Ltc (ms)	[L,PI,PO]	[fr,wr]	lut	Ltc (ms)
CL0	[3,224,224]	[64,3]	[3,3,8]	0.680	6.024	[3,3,32]	[3,1]	0.469	1.004
CL1	[64,224,224]	[64,64]	[3,3,8]	0.680	132.467	[3,11,13]	[3,1]	0.699	15.054
CL2	[64,112,112]	[128,64]	[3,8,3]	0.680	64.731	[3,11,13]	[3,1]	0.699	7.527
CL3	[128,112,112]	[128,128]	[3,3,8]	0.680	129.455	[3,11,13]	[3,1]	0.699	15.054
CL4	[128,56,56]	[256,128]	[3,3,8]	0.680	64.728	[3,11,13]	[3,1]	0.699	7.527
CL{5,6}	[256,56,56]	[256,256]	[3,3,8]	0.680	127.455	[3,11,13]	[3,1]	0.699	15.053
CL7	[256,28,28]	[512,256]	[3,8,3]	0.680	64.352	[3,13,11]	[3,2]	0.699	7.370
CL{8,9}	[512,28,28]	[512,512]	[3,3,8]	0.680	128.702	[3,11,13]	[3,2]	0.699	14.740
CL{10,11,12}	[512,14,14]	[512,512]	[3,3,8]	0.680	32.178	[3,11,13]	[3,2]	0.699	3.685
FCL0	25088	[4096,25088]	[4,-,-]	0.038	128.445	[16,-,-]	[-,1]	0.039	21.408
FCL1	4096	[4096,4096]	[4,-,-]	0.038	20.966	[16,-,-]	[-,1]	0.039	3.494
FCL2	4096	[1000,4096]	[4,-,-]	0.038	5.119	[16,-,-]	[-,1]	0.039	0.853

**Table 4:** Resource consumption and processing latency of each layer.

Layer	XC7A100T				ZU15EG			
	[L,PI,PO]	lut	Ltc_e	Rcfg	[L,PI,PO]	lut	Ltc_e	Rcfg
A_CL0	[11,3,2]	0.673	0.112%	1.725	[11,3,13]	0.716	0.134%	6.675
A_CL1	[5,1,14]	0.715	0.023%	1.989	[5,5,17]	0.709	0.018%	6.664
A_CL{2,3,4}	[3,1,24]	0.725	0.025%	2.055	[3,13,11]	0.733	0.021%	6.819
A_FCL{0,1,2}	[4,-,-]	0.039	≈0	-	[24,-,-]	0.040	≈0	-
V_CL0	[3,3,8]	0.734	0.146%	-	[3,3,32]	0.498	0.172%	6.385
V_CL{1-12}	[3,3,8]	0.734	0.028%	2.122	[3,11,13]	0.719	0.047%	6.714
V_FCL{0,1,2}	[4,-,-]	0.039	≈0	-	[24,-,-]	0.040	≈0	-

**Table 5:** Comparison to previous works.

	AlexNet						VGG16					
		**[6]**	**[23]**	**[26]**	**Ours**	**Ours**		**[27]**	**[20]**	**[19]**	**Ours**	**Ours**
Device	1080ti	7VX485T	ZU9EG	KU115	7A100T	ZU15EG	1080ti	7Z045	KU060	7VX980T	7A100T	ZU15EG
Precision	32b fp	32b fp	3/16b fx	16b fx	32b fp	32b fp	32b fp	16b fx	16b fx	8/16b fx	32b fp	32b fp
Frequency (MHz)	1481	100	200	230	200	300	1481	150	200	150	200	300
Logic Cell (K)	-	485.7	600	1451	101.44	747	-	350	726	979.2	101.44	747
DSP	-	2800	2520	5520	240	3528	-	900	2760	3600	240	3528
Bandwidth (GB/s)	484.4	4.5	-	-	3.125	18.75	484.4	4.2	12.8	12.8	3.125	18.75
Latency (ms)	0.733 (C) 1.145 (O)	21.62 (C)	-	0.59 (O)	48.556 (C) 123.870 (O)	18.675 (C) 37.886 (O)	5.304 (C) 6.476 (O)	163.42 (C) 224.60 (O)	101.15 (O)	-	1070.605 (C) 1227.257 (O)	130.562 (C) 163.031 (O)
Power (W)	250	18.61	19.6	38.79	1.617	3.401	250	9.63	25	14.36	1.821	3.729
Performance (GOP/s)	1788.722 (C) 984.409 (O)	61.62 (C)	957.4 (C)	2411.01 (O)	29.239 (C) 12.095 (O)	245.71 (C) 105.986 (O)	5799.397 (C) 4749.846 (O)	187.80 (C) 136.97 (O)	310 (C) 266 (O)	1000 (O)	28.731 (C) 25.107 (O)	247.711 (C) 217.625 (O)
Power Eff (GOP/s/W)	7.155 (C) 3.938 (O)	3.310 (C)	48.85 (C)	62.16 (O)	18.082 (C) 7.478 (O)	72.278 (C) 31.163 (O)	23.176 (C) 18.999 (O)	19.50 (C) 14.22 (O)	12.4 (C) 10.64 (O)	69.64 (O)	15.778 (C) 13.787 (O)	66.074 (C) 58.360 (O)
Logic Cell Eff (GOP/s/Kcells)	-	0.127 (C)	1.596 (C)	1.662 (O)	0.288 (C) 0.119 (O)	0.329 (C) 0.142 (O)	-	0.537 (C) 0.391 (O)	0.427 (C) 0.366 (O)	1.021 (O)	0.283 (C) 0.248 (O)	0.332 (C) 0.291 (O)
DSP Eff (GOP/s/DSP)	-	0.022 (C)	0.381 (C)	0.437 (O)	0.122 (C) 0.050 (O)	0.069 (C) 0.030 (O)	-	0.209 (C) 0.152 (O)	0.112 (C) 0.096 (O)	0.278 (O)	0.120 (C) 0.105 (O)	0.070 (C) 0.062 (O)

## Data Availability

The source code is available at https://github.com/xuyh55/Flare (accessed on 2 March 2024).

## Abbreviations

The following abbreviations are used in this manuscript:

CNN	Convolutional Neural Network
FPGA	Filed Programmable Gate Array
MAC	Multiply ACcumulate
ASIC	Application Specific Integrated Circuit
HLS	High Level Synthesis
DSE	Design Space Exploration
CL	Convolutional Layer
FCL	Fully Connected Layer
GEMM	General Matrix Multiplication
FFT	Fast Fourier Transform
PE	Processing Element
PI	Parallelism levels of Input channel
PO	Parallelism levels of Output channel
DPC	Data Per Clock
